# Editorial: The Dual-Use Dilemma for Biomimicry

**DOI:** 10.3389/fmolb.2022.915663

**Published:** 2022-05-19

**Authors:** Samar Damiati, Rami Mhanna, Shakil A. Awan, Rimantas Kodzius, Bernhard Schuster

**Affiliations:** ^1^ Department of Chemistry, College of Sciences, University of Sharjah, Sharjah, United Arab Emirates; ^2^ Biomedical Engineering Program, American University of Beirut (AUB), Beirut, Lebanon; ^3^ Wolfson Nanomaterials and Devices Laboratory, School of Engineering, Computing and Mathematics, Faculty of Science and Engineering, University of Plymouth, Plymouth, United Kingdom; ^4^ Faculty of Medicine, Ludwig Maximilian University of Munich (LMU), Munich, Germany; ^5^ Department of Nanobiotechnology, Institute for Synthetic Bioarchitectures, University of Natural Resources and Life Sciences (BOKU), Vienna, Austria

**Keywords:** biomimicry, synthetic cells, bioengineering, diagnostic-therapeutic platforms, drug vehicles

**FIGURE 1:**
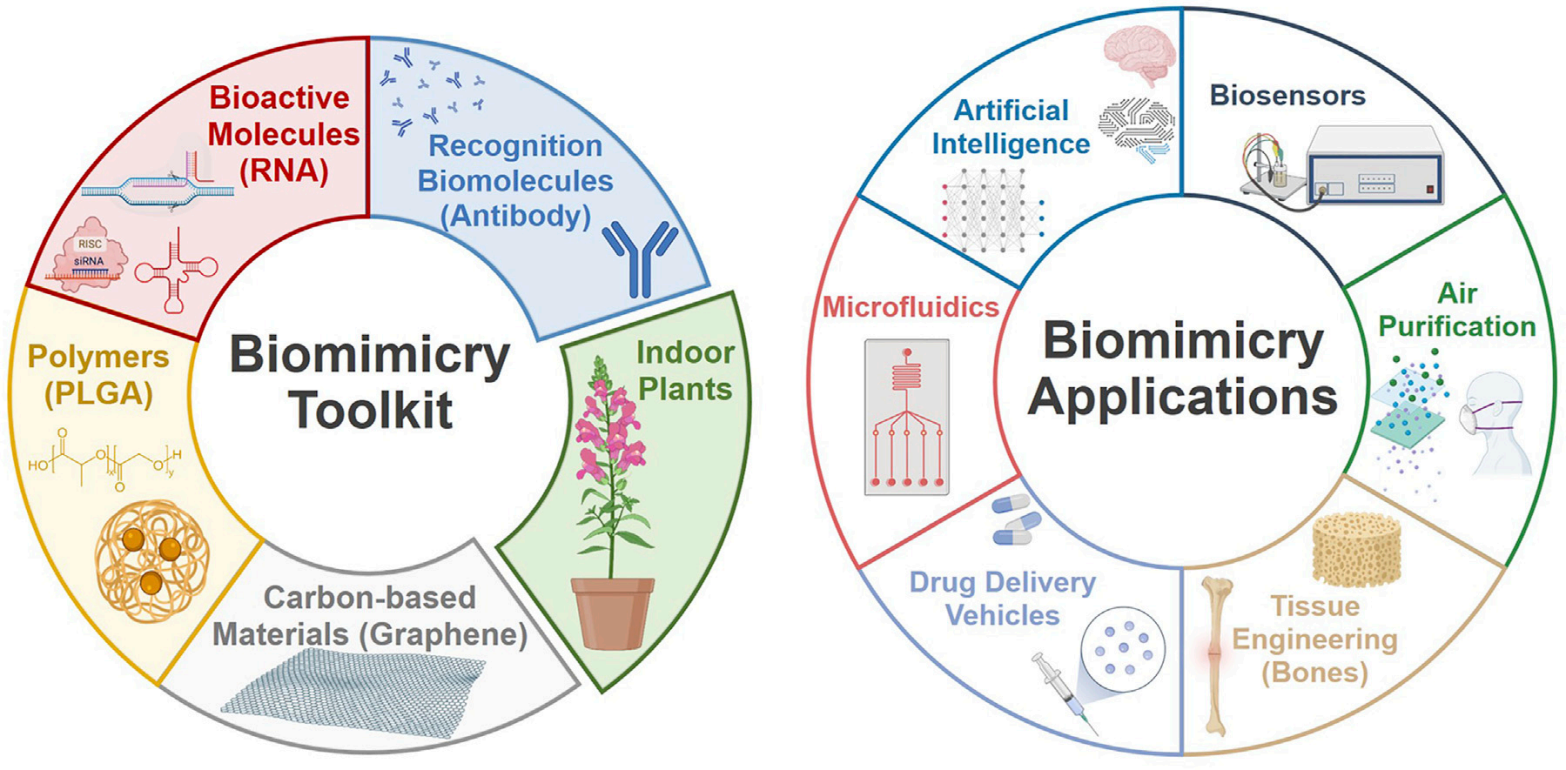
Biomimicry: tools and applications (Created with Biorender.com).

The biomimetic fabrication of 3D biology models confers design freedom across various applications. Taking inspiration from nature and using nature as a toolbox have helped in the production of effective and versatile tools that are integrated with modern technology principles and, importantly, perform quite similarly to natural living systems. Exploiting nature’s design principles can contribute to the improvement of the understanding and development of creative products in both fundamental research and industrial applications (Dicks, 2016; Orapiriyakul et al., 2018; Damiati, 2020). To address the significant heterogeneity among bio-inspired designs, this research topic collected articles that reflect the progress in the field. The topics range from the manufacture of graphene field-effect transistor (GFET) biosensors for the improvement of sensing technology, to combining AI and microfluidic technologies for the rapid fabrication of drug vehicles, to recent findings regarding the mimicry of bone’s natural behavior in bone tissue engineering, and to eco-friendly solutions for indoor air purification fighting COVID-19 (Figure 1). All the recent developments and significant accomplishments bring new opportunities to improve our quality of life.

Alzheimer’s disease is the most common degenerative disorder in the world. The combination of naturally occurring minerals in nature such as graphene and capturing biomolecules such as antibodies offers a new opportunity for developing simple diagnostic devices with remarkable performance. The challenging task in developing a biosensor is building a sensing matrix that allows the sensitive and selective capture of biomarkers with low limits of detection. Bungon et al. developed a promising GFET biosensor for sensing and targeting clusterin, a prominent protein biomarker of Alzheimer’s disease. Considering the remarkable properties of graphene such as its high carrier mobility, current carrying capacity, thermal conductivity, optical properties, and mechanical stability, the material has been employed in FET biosensing platforms. The quantitative responses of the GFET sensors were determined using direct current 4-probe electrical resistance measurements, which demonstrated a limit of detection for the biosensors of four fM.

Drug development is a time consuming and expensive process. Many factors may delay drugs’ approval and their availability on the market. For example, the limited utilization of several non-steroidal anti-inflammatory drugs (NSAIDs) is attributed to their poor water solubility. Hence, controlled-release carriers to improve solubility and increase bioavailability are highly demanded. The encapsulation of NSAIDs within a polymeric matrix would allow the sustained and controlled release of the loaded drugs. Furthermore, with the continuous development of artificial intelligence (AI) technologies, the frontiers for polymeric particles and drug-delivery carriers have been greatly advanced. To speed up a drug candidate’s progress, combining machine learning and microfluidic technologies, alongside using biodegradable polymers, represents a promising means for generating drug-loaded polymeric microparticles (MPs). Damiati and Damiati developed a strategy based on machine learning using artificial neural networks (ANNs) as an in silico tool, a 3D flow-focusing microfluidic chip as a simple device to produce MPs, and poly (D,L-lactide-co-glycolide) (PLGA) as a matrix material for the encapsulation of indomethacin (IND), a type of NSAID. The developed strategy represents a powerful tool for predicting and generating size-tunable PLGA MPs. The ability to control the particle size and particle-size distribution is critical in the pharmaceutical industry. The generation of small sizes and narrow size distributions is usually favorable for obtaining final particles with high stability and long shelf-lives, as well as enhancing the bioavailability of poorly soluble drugs.

Mimicking natural bone for bone grafting has been achieved by taking advantage of the cell’s molecular machinery. Many researchers focus on the development of gene therapy and understanding the mechanisms of bone remodeling to create functional bone tissue that can cure bone defects. In this regard, Damiati and El-Messeiry review combinations of cells, biomaterial scaffolds, and bioactive molecules such as RNA coatings. Different types of RNA including messenger RNA (mRNA), RNA interference (RNAi) molecules, small interfering RNA (siRNA), and long non-coding RNA (lncRNA) play roles in cellular *ex vivo* and *in vivo* bone regeneration. Authors review the possibility of seeding mammalian cells and synthetic RNA molecules onto natural or synthetic scaffolds such as polymers and metals to fabricate osteogenic implants. The advantages and limitations of the RNA-based scaffolds are also discussed. Authors highlight using the CRISPR-based genome editing technology to guide RNA scaffolds in bone regeneration. CRISPR/Cas9 represents a promising tool for elucidating various mechanisms of osteogenesis and bone healing in a cost- and time-efficient manner, as well as providing a new strategy for controlling bone infection.

Due to expectations that the severe acute respiratory syndrome coronavirus-2 (SARS-CoV-2), causing coronavirus infectious disease (COVID-19), will continue to spread and might become a common seasonal infection, there is global interest in fabricating filters that can remove the virus from the air. As part of ongoing efforts to find several techniques that can be applied to the bio-decontamination of air, El-Tanbouly et al. review the utilization of indoor plants as an eco-friendly tool to remove air pollutants and habituated airborne microbes. In comparison to filtration and radiation, which are expensive and not feasible for home use, indoor plants are a cost-efficient means of indoor air purification that can be easily adapted to an environment, without special requirements. Phytoremediation technologies commonly use plants to remove hazardous contaminants from environments. Besides the fact that indoor plants are natural air filters that purify air through different mechanisms involving absorption, filtration, dilution, and precipitation, plants have the ability to release small amounts of secondary metabolites and their derivatives, such as polyphenols and alkaloids, into the air. The released compounds show antimicrobial actions and can efficiently interact with the surrounding environment, which may contain airborne microbes. Moreover, regulating humidity in the indoor environment using indoor plants may be useful for reducing SARS-CoV-2 transmission and, thus, in fighting COVID-19.

In summary, the collection of articles in this Research Topic highlights the diverse applications of synthetic biology. These studies offer valuable insights to nature inspired technology and will inspire scientists to extend the future of biomimetic products and to deploy biomimetics in various fields.
